# Perplexing issues for convalescent immune plasma therapy in COVID-19

**DOI:** 10.14744/nci.2021.73604

**Published:** 2021-11-15

**Authors:** Oner Ozdemir

**Affiliations:** Division of Allergy and Immunology, Department of Pediatrics, Sakarya University Training and Research Hospital, Sakarya, Turkey

**Keywords:** Convalescent immune plasma, COVID-19, neutralizing antibody titer, SARS-CoV-2

## Abstract

Convalescent immune plasma (CIP) therapy in coronavirus disease 2019 (COVID-19) is presently a trendy choice of treatment. On March 24, 2020, the United States Food and Drug Administration approved of CIP treatment for seriously ill COVID-19 patients as an emergency investigational new drug. The precise mechanisms of action for CIP in COVID-19 have not yet been undoubtedly recognized. However, earlier research demonstrated that the main mechanism of CIP such as in other viral infections is viral neutralization. Systematic reviews and meta-analyses of the CIP transfusion in severe infectious diseases have shown that CIP has some beneficial effects and it is a harmless process to cure infectious diseases early after symptom beginning. It is suggested that SARS-CoV-2 neutralizing antibody titers in CIP should be ideally higher than 1:320, but lower thresholds could also be useful. The suggested minimum dose for one individual is one unit (200 mL) of CIP. The second unit can be given 48 h succeeding the end of the transfusion of the first unit of CIP. Moreover, CIP can be applied up to a maximum of three units (600 mL). CIP could be administered in other systemic diseases, viral infections coincidentally associated with SARS-CoV-2 infection, as well as other therapeutic approaches for COVID-19. There are generally no serious adverse events described from CIP transfusion in these recipients. CIP may have a significant role as one of the therapeutic modalities for various viral infections when enough vaccines or other specific therapeutic agents are not on hand.

**C**onvalescent immune plasma (CIP) therapy in coronavirus disease 2019 (COVID-19) is presently trendy choice of treatment [1–4]. On March 24, 2020, the United States Food and Drug Administration (FDA) approved of CIP treatment for critically ill COVID-19 patients as an emergency investigational new drug [2]. In this article, first CIP therapy and its mechanisms are described and later dose, frequency, timing, administration with other therapeutics and in systemic diseases, its biological safety, adverse effects, and last pearls-pitfalls of the CIP transfusion will be discussed.

## What is CIP?

CIP is obtained from the plasma part or the whole blood of recuperated COVID-19 patients, which includes proteins known as antibodies produced by the immune system to battle with the SARS-CoV-2 infection. Plasma is the liquid part of blood and these antibodies in plasma can be collected by means of two methods (plasmapheresis or whole blood donation) and later utilized to treat other COVID-19 patients by CIP transfusion that is safe and has known a few side effects [3, 4]. (The answers of three key questions for CIP transfusion are given in Table 1).

**Table 1.** The answers of three key questions for CIP transfusion

What is known about the topic?	What is new?	What are the future key questions for future work on the topic?
Passive antibody therapy has begun ahead of the 20^th^ century	CIP best works before 14 days of hospitalization	What circumstances in the patient make CIP transfusion possible treatment alternative?
CIP was found to be effective in Ebola and SARS-CoV-1 infections	ADE is suspected to be life-threating complication	What will be the exact dose of CIP?
TRALI and TACO are known to be a transfusion-related complications		

CIP: Convalescent immune plasma; TRALI: Transfusion-related acute lung injury; TACO: Transfusion-associated circulatory overload; ADE: Antibody-dependent immune enhancement.

Two procedures of donating CIP are following: First of the two methods, plasmapheresis is the typical process by which plasma is separated from whole blood and collected. This utilizes a machine which differentiates the four elements of whole blood (red blood cells, white blood cells, platelets, and plasma) but gathers only the plasma, and gives the rest back to the donor. Giving a plasma only donation continues 90 min from start to finish and delivers a greater amount (2 units) of plasma than obtaining plasma from whole blood. Second, when you give CIP through whole blood, you as a donor give a normal blood donation, but it is processed differently. Giving CIP by blood donation lasts an hour and results in two units of blood products (one unit of CIP and one unit of red blood cells) [5].

## Mechanisms of Actions and Other Beneficial Effects of CIP Transfusion

The precise mechanisms of action for CIP in COVID-19 have not yet been undoubtedly recognized. However, earlier research demonstrated that the main mechanism of CIP such as in other viral infections, for example, Ebola and respiratory syncytial virus is viral neutralization [6]. In the incident of SARS-CoV-2, the predicted mechanism by which passive antibody/CIP treatment would confer defense is viral neutralization. Neutralizing antibodies provided by CIP can control the virus load. Nevertheless, the existence of non-neutralizing antibodies attached to the causative agents might also be useful and they may also add benefit into therapy and/or prophylaxis and increase rescue [6, 7]. Thus, primarily, the obvious mechanism relates to the fact that antibodies from CIP transfusion can overwhelm viremia through neutralization. Other mechanisms such as antibody-dependent cellular cytotoxicity, complement activation, and/or phagocytosis might contribute as well [7].

**Highlight key points**

• CIP transfusion can assist to impede viral spread and improve survival in COVID-19 cases, especially having pulmonary insufficiency.
• CIP therapy should be started to COVD-19 cases at an early phase of SARS-CoV-2 infection and should probably be utilized in potentially seriously ill individuals.
• There were generally no serious adverse events described from CIP transfusion in these recipients.

On the whole, the CIP utilization can also deliver an immunomodulatory role through improvement of macrophage stimulation and systemic hyperinflammation or “cytokine storm” as well. The given antibodies can adjust hyperinflammatory response and this can be ideally accomplished during the initial reaction, even at the asymptomatic phase [7]. For instance, there has been one reported study showing that CIP therapy could lessen serum cytokine response [8]. It has also been thought that aside from the direct anti-viral properties, other healing plasma elements from recovering donors can produce other valuable activities, such as reinstating procoagulant or antifibrinolytic activity, preventing excess vascular leakage, and repairing the endothelium glycocalyx [9, 10].

## Course of the Disease and Development of Neutralizing Antibodies

Viremia summits during the 1^st^ week of most viral infections such as in SARS, and since the primary immune reaction classically matures by day 10–14, pursued by viral clearance or more possibly by cytokine storm that could be fatal [8, 11]. Cao and Shi investigated SARS-CoV-2 neutralizing antibody titers (NATs) in 56 individuals recuperated from SARS-CoV-2 infection. Their results showed that SARS-CoV-2 IgG and NAT reached the peak at 4 months and then waned; decreasing untraceable levels in 25.6% (IgG) and 16.1% (NAT) of study participants at 36 months [9].

## Effectiveness of CIP Transfusion

Systematic reviews and meta-analyses of the CIP transfusion in severe infectious diseases have showed that CIP has some beneficial effect and it is a harmless process to cure infectious diseases early after symptom beginning. CIP is a theoretically effective therapy and can function as an encouraging saving choice for stark SARS-CoV-2 infections [12]. A review by Yiğenoğlu states that CIP seems to be a harmless and most likely efficient therapy for critically ill COVID-19 cases until the vaccines or new therapeutic modalities are developed [6]. In a systematic review of the current immunotherapies of COVID-19, including CIP therapy in 33 cases from 24 studies, it presented clinical data of results. It seemed that immunotherapy together with other usual cares could be a useful and safe strategy to adjust the immune system and improvement of clinical results [13]. A rigorous systematic review included total 10 studies comprising of a mix of case reports, case series, observational studies, and randomized control trials. It stated that it was found to be hard to depict a definitive conclusion bearing in mind the restrictions in the design of current research. However, the findings demonstrated that CIP transfusion makes remarkable healing in cases’ clinical symptoms, radiological and biochemical parameters [14]. In another systematic review by Rajendran et al. [15], they evaluated 27 patients (male: 15, female: 12) receiving CIP from five different studies. The age of the cases in the studies was ranging from 28 to 75 years. They concluded that CIP transfusion in COVID-19 seemed to be safe, clinically efficient, and decreased mortality dependent on the limited evidence.

In a prospective study of 10 severe patients by Duan et al. [16], they showed that CIP transfusion was well tolerated and could potentially get the clinical and radiological results better through neutralizing viremia in severe COVID-19 cases. In addition, CIP has been demonstrated to be related with decreasing ventilator necessities in cases with both severe and life-threatening diseases, but seems to be most favorable when given promptly in the progression of disease when cases encounter the conditions for severe disease [17]. Similarly, in a study by Erkurt et al. [18], one unit of CIP (200 ml) transfusion was administered to 26 COVID-19 cases in ICU. COVID-19 cases did not need mechanical ventilation, CIP therapy was thought to be a curative therapeutic option if given in early course of the disease. Furthermore, the data of severe or critically ill COVID-19 cases who had CIP transfusion together with the antiviral therapy (n=888) and matched severe or critically ill COVID-19 cases did not have CIP at 1:1 ratio (n=888) were retrospectively evaluated. Length of stay in ICU, ratios of mechanical ventilation and vasopressor need were significantly lower in CIP group compared with the control. CIP administration 20 days after the COVID-19 diagnosis was related with a higher ratio of mechanical ventilation support. CIP therapy appeared to be effective, especially given early, for a better course of COVID-19 in severe and critically ill cases [19]. As a result of various systematic reviews and trials, CIP seems to be effective therapeutic option, especially in critically ill COVID-19 cases, when it is applied at the right time and exact dose.

However, conflicting reports are still ongoing in current literature. For instance, in a randomized trial by Simonovich et al. [20], CIP was given to hospitalized adult cases of severe COVID-19 pneumonia. At the 30^th^ day, no considerable discrepancy was found between the CIP and the placebo groups for regarding clinical outcomes. Overall mortality was 10.96% in the CIP group and 11.43% in the placebo group, for a risk variation of −0.46 percentage points. As a result, no noteworthy discrepancy was detected in clinical picture or general mortality between patients transfused with CIP and those who took placebo.

## Insufficiency of CIP Transfusion

Nevertheless, there have been some reports indicating insufficiency of CIP therapy in recent literature. Some authors thought that research of non-COVID-19 severe respiratory viral infections deliver ancillary, very low-quality proof that increases the likelihood that CIP has insignificant or no advantage in the therapy of COVID-19 [21]. There have been also some case reports of non-optimal effectiveness of CIP transfusion and hydroxychloroquine combination for treating COVID-19 [22]. The neutralizing antibodies existed in CIP did not seem to affect the cytokine storm caused by SARS-CoV-2 infections. Unsuccessful modification of cytokine storm syndrome was shown in some studies [23]. Furthermore, the second Cochrane meta-analysis in 2020 by Piechotta et al. [24] evaluated 20 studies (one randomized controlled trial, three non-randomized controlled studies of interventions, and 16 non-randomized non-controlled studies of interventions) with 5443 participants, of who 5211 received CIP. In contrast, the authors concluded that they were very indeterminate whether CIP is favorable for hospitalized COVID-19 patients.

## What is the Application Dose and Frequency of CIP Transfusion?

Conditions of being recipient have been described in detail in some regulatory/position articles [25]. There have been almost 10 parameters (persistent fever [≥5 days], various criteria of respiratory failure, need for vasopressor and rapid clinical deterioration and those with dire prognostic parameters, etc.) defined for the CIP transfusion [26]. CIP can be gathered by apheresis or whole blood donation. Apheresis accumulates 200–800 mL of CIP that can be separated into 1–4 units before freezing. Since there is lack of verification on its dosing and effectiveness, the quantity of viral antibodies administered to each case is indefinite and not identical, which may cause to dissimilarities in clinical effect. The least efficient dose, and whether that is associated with a precise NAT, is presently unidentified [7]. In the end, the success of the CIP treatment seems to vary based on the type of microorganism and therapy protocols (e.g., timing, volume, and application dose).

In earlier utilization of CIP therapy in SARS, 5 mL/kg of CIP at a titer of 1:160 was administered. A quarter or half of the therapeutic dose was preventatively utilized in the past studies. Consistent with linear proportionality, 3.125 mL/kg of CIP with a titer of >1:64 would supply a comparable immunoglobulin level to one-quarter of 5 mL/kg CIP with a titer of 1:160 [6]. A study trying CIP with a serum antibody titer of >1:640 in SARS therapy demonstrated that severe cases survived after the therapy [27]. In previous research from China, two successive 200–250 mL of ABO matched CIP were administered in one investigation, but only one 200 mL dose with anti-SARS-CoV-2 titer >1:640 was tried in another [7]. The existence of sufficient levels of anti-SARS-CoV-2 NAT is advised (a titer of ≥1:320 is suggested only for cases influenced by primary or acquired [as well as cases used B-cell depleting monoclonal antibodies] immunodeficiency) [28]. Shen et al. [29] described a case series of five seriously ill patients, all utilizing CIP including SARS-CoV-2 antibody titer >1:1.000 and a NAT higher than 1:40, applied between day 10 and 22 of admission. Duan et al. [16] reported a series of 10 serious COVID-19 cases, all taking a 200 mL CIP with high NAT (>1:640) at a median of 16.5 days. In a study, among evaluated 64 CIP donors, donors (except one) had a spike receptor-binding protein (S-RBD)-specific IgG titer ≥1:320. If any donor has a titer ≥1:160 for S-RBD-specific IgG antibody by EIA method or equivalent with other methods, they meet the CIP quality control conditions depending on the Chinese national directives for CIP [30].

FDA and European commission suggest that SARS-CoV-2 NATs should favorably be at ≥1:160 or ≥1:320, respectively; as an enrollment criterion for donor collection. If such a suitable unit is not accessible, both regulatory commissions permit for lower titers (e.g., ≥1:80) [7]. Turkish CIP regulatory guidelines also accept the lower (≥1:80) titers [25]. CIP has been incorporated as a therapeutic choice in the Chinese COVID-19 therapy guiding principles and a viral titer of 1:160 has been accepted as a quality control marker [30]. It is suggested that SARS-CoV-2 NATs should be higher than 1:320, but lower thresholds could also be useful [31].

The suggested minimum dose for one individual is one unit (200 mL) of CIP. Second unit can be given 48 h succeeding the end of the transfusion of the first unit of CIP. Moreover, CIP can be applied up to a maximum of three units (600 mL) [25].

## Time of Collection For CIP

Who can donate CIP has been also very well defined in the recent literature. The FDA and other regulatory agencies have approved the use of CIP from individuals with recovered COVID-19 by different guidelines [32]. The two key clinical and laboratory criteria are basically described as the proof of SARS-CoV-2 infection by clinical and/or laboratory confirmation, and 14–28 days of resolution of symptoms before donation [32]. According to the FDA guidelines, CIP is gathered from patients whose plasma includes anti-SARS-CoV-2 antibodies and who convene all donor eligibility necessities. After testing for pertinent transfusion-transmitted infections (TTIs) checked, CIP is accumulated from cases who meet the following qualifications: (i) Verification of COVID-19 by laboratory testing, (ii) absolute recovery of symptoms no <14 days ahead of the donation, and (iii) male donors, female donors who have never been heavy with child, or female donors checked as negative for anti-HLA antibodies [33]. In line with the “Clinical Treatment of Convalescent Plasma for COVID-19 (trial edition 2)” reported by the National Health Commission of China, the donor’s plasma should be gathered 3 weeks after the beginning of disease [34]. CIP contribution won’t harm the donor when the patient has been discharged from the hospital for 14 days [35]. CIP from donors who have recuperated and who are at week 12 after disease start is anticipated to be more useful [8]. The most favorable time to gather the CIP requires to be clarified in further randomized controlled studies.

## What is the Best Time for CIP Transfusion

Should it be earlier (<10 days) or is late (>10 days of initial symptoms) transfusion? The best timing of applying CIP in COVID-19 cases has to be cautiously thought.

From the knowledge in other viral diseases, CIP ought to be utilized early and more effective in earlier stage of the disorder, ahead of the hyperinflammatory syndrome, and at the peak of producing of endogenous IgM and IgG antibodies. Indeed, the beneficial effect of CIP on COVID-19 is detected by the level of NAT. An investigation on SARS showed that the specific IgG started to enhance approximately week 3 after COVID-19 beginning and maximized at week 12. Moreover, studies on SARS seem to have verified this presumption as well [8, 36]. Early CIP application in COVID-19 cases is also considered to avert innate immune cell movement and thwart pulmonary injury. CIP should hypothetically be more valuable when administered early during disorder (i.e., before day 14, or through the viremia and seronegative period) [37]. Likewise, cases to whom CIP given earlier (before day 14 of symptom beginning) were considerably more prone to be discharged earlier than day 22 (58% vs. 16%) and inclined toward lesser fatality (6.3% vs. 21.9%, p=0.08) than those who started therapy following day 14 [38]. Yet, a study demonstrated that, when CIP infusions were begun on the day of diagnosis or up to 2 days afterward, the mortality risk between day 3 and day 16 was slight [9, 10].

It has not been advised to administer after 14 days of the disease’ start as well as before the beginning of cytokine storm (exaggerated hyperimmune attacks) [25, 39]. In a regulation distributed by FDA, CIP is advised to be given between 7 and 14 days in COVID-19 cases that fulfill with the identified criteria FDA [40].

## Biological Safety of CIP Transfusion

There is only very low certainty evidence for safety of CIP in COVID-19 treatment. There is a hypothetical risk of spreading SARS-CoV-2 by transfusion, particularly with the existing deficiency of donor selection for frequent respiratory viruses. In a new research, four asymptomatic out of 2430 selected platelet and whole blood donors had detectable SARS-CoV-2 RNA in their blood. Nevertheless, demonstrable RNA did not automatically indicate contagiousness. To the best of authors’ knowledge, there has never been a description of respiratory virus spread thru blood transfusion; however, this requires to be evaluated by continuing scrutiny [41].

Liquid plasma could be stored, within 24 h subsequent to blood donation, at 1–6°C for up to 40 days, and plasma frozen at ≤−18°C can be stored for up to 12 months. Inactivation of plasma causative agents should be verified to minimize the chance of transfusion spread contagious diseases and to exclude the likely risk of SARS-CoV-2 superinfection [31, 33].

## CIP Administration in Other Systemic Diseases and Viral Infections Associated with COVID-19

The CIP could be a potential lifesaving option to treat critical COVID-19 cases with underlying diabetes or hepatic dysfunction [42]. In a renal transplant recipient with severe clinical manifestation and difficult complications, CIP was helpful treatment in COVID-19 [43]. CIP treatment was demonstrated to be useful in a myelodysplastic COVID-19 case with systemic tuberculosis [44]. A 6-year-old girl with severe COVID-19-related severe aplastic anemia, in whom SARS-CoV-2 infection was effectively eradicated after CIP given [45]. A SARS-CoV-2 infected X-linked agammaglobulinemia case showed rapid recovery of after CIP administered [46]. Severe refractory COVID-19 cases were observed to respond to CIP transfusion very well in a case series. Two male (a 46 and 56 years old) patients worsened in spite of palliative care and antiviral treatment, they began to recover with CIP transfusion both clinically and radiologically [47].

## CIP Administration with Other Therapeutic Approaches In COVID-19

Immediate recovery was reported after CIP transfusion for persisting COVID-19 following therapeutic lymphocyte depletion by combined rituximab and bendamustine treatment for lymphoma [48]. In the management of severely ill COVID-19 patients, a synergistic role of CIP administration and mesenchymal stem cells was suggested [49]. Efficiency of early therapeutic plasma exchange by CIP for severe COVID-19 as replacement fluid was shown. For effective cytokine clearance, 1.5 volume of case’s plasma should be discarded. This approach was suggested to be thought as a therapeutic alternative in severe COVID-19 cases within the 1^st^ week of symptom beginning [50]. In those earlier studies, CIP was transfused on average 15.7 and 21 days following symptom beginning. Bearing in mind the pathophysiological course of the disorder, this timing is quite delayed for immunomodulatory effect [50].

## Adverse Effects of CIP Transfusion

The first Cochrane meta-analysis of described case series in 2020, the adverse effect ratios were found to be very low [51]. Furthermore, a study demonstrated a low rate of severe adverse effects during the first 4 h of infusion (<1%). Risks related with CIP are prone to be the identical as those with regular plasma, consisting of mild (e.g., allergic and febrile) to impending life-threatening events (e.g., transfusion-related acute lung injury [TRALI], transfusion-associated circulatory overload [TACO], anaphylaxis, etc.) [3].

## Common or Well-Known Side Effects

There were generally no serious adverse events described from CIP transfusion in these recipients.

Similar to any other blood product infusion, there are some widespread, expected, or known adverse effects that are also relevant to CIP treatment. By following meticulous modern blood banking techniques and transfusion safety measures, the cumulative risk of any life-threatening reactions is <1% [52]. The most frequent adverse effect of CIP treatment are transfusion-associated reactions, including chills, fever, serum sickness, anaphylactic reactions, TRALI, TACO and hemolysis, etc. In the meantime, the risk of TTIs, such as human immunodeficiency virus, hepatitis B virus, hepatitis C virus, and syphilis, should not be ignored [7, 25, 26]. To diminish the adverse events of infusion and advance the clinical curative effect, the cases could be given promethazine or dexamethasone before CIP transfusion [35].

In a study of 20,000 hospitalized cases treated with CIP, the frequency of all severe adverse effects was low; they involved infusion events (<1%), thromboembolic or thrombotic events (<1%), and cardiac events (~3%). Particularly, the most of the thromboembolic or thrombotic and cardiac events were thought not to be associated with CIP transfusion [52].

A study analyzed events after transfusion of ABO matched human CIP in 5000 hospitalized adults with severe or life-threatening COVID-19, with 66% in the intensive care unit. The frequency of all severe adverse effects in the first 4 h following infusion was <1%, involving fatality rate (0.3%). Of the 36 described severe adverse effects, there were 25 described effects linked to severe adverse effects, comprising fatality (n=4), TACO (n=7), TRALI (n=11), and severe allergic events (n=3). Nevertheless, only 2/36 severe adverse effects were thought as certainly associated with the CIP by the physician. After CIP transfusion in 5000 cases, the frequency of severe adverse effects was <1% and the 7-day frequency of fatality was 14.9% [53].

## TRALI of CIP Transfusion

The most significant worry among physicians during the CIP utilization is TRALI. It is described as an acute respiratory distress syndrome that takes place within 6 h of blood received [54]. Some clinicians have indistinct distress about TRALI when transfusing ABO mismatched CIP, but TRALI is infrequently related to ABO mismatch [55]. To diminish the risk of TRALI, it is advised to avoid from formerly pregnant female donors including who had abortions. Whereas the TRALI possibility is usually <1/5.000 unit transfusion, it is of specific concern in severe SARS-CoV-2 infections with pulmonary disease having a risk for TRALI, given the possible inducement of the pulmonary endothelial damage [31]. HLA antibody test is customarily performed as a preventive procedure against TRALI. Pre-donation HLA antibody test might be valuable in parous females since up to a third of women who report having been formerly pregnant have HLA antibodies. In nations where HLA and human neutrophil antigen (HNA) antibody testing are unaffordable, eligibility to donate CIP might be limited to males and nulliparous females [3].

## TACO

This might take place in as many as 12% of at-risk individuals [55]. It is even greater in elderly COVID-19 cases with acute pulmonary damage that is being maintained with mechanical ventilation. TACO might be particularly pertinent for those patients with viral myocarditis. The “dose” of plasma being thought for CIP is well within the range related to TACO in non-COVID-19 cases [56]. Noteworthy, risk elements for TACO (e.g., cardiopulmonary disease, advanced age, kidney impairment, etc.) are mutual by those at risk of COVID-19, underlining the caution for given volume [31]. Especially, in stern COVID-19 cases, vascular permeability is augmented by cytokines; therefore, it is essential to be cautious regarding volume overload [55].

TRALI and TACO are predominantly worrying in serious COVID-19 related to circumstantial acute pulmonary damage and probable priming of the damage in lung endothelium. This emphasizes the significance of CIP donor collection to circumvent high-risk donors. Accordingly, the European Union program necessitates CIP donors without a history of any transfusion record and female donors who have never been heavy with child, or are tested and detected negative for human leukocyte/platelet/neutrophil antigen antibodies (anti-HLA/HPA/HNA, respectively) utilizing a certified test [3]. Pre-treatment (e.g., acetaminophen and diphenhydramine) to abate transfusion-related events might be thought, as required, or if the case had formerly required pre-medication for blood transfusions. Whatever quantity of CIP is utilized, cases at risk of TACO (short, underweight, elderly, identified or assumed kidney or cardio-respiratory insufficiency) should be received CIP therapy gradually – at a speed as little as 1 mL/kg/hour and carefully observed during the transfusion [4, 7].

## Antibody-Dependent Immune Enhancement (ADE) of Infection

**Figure 1.**
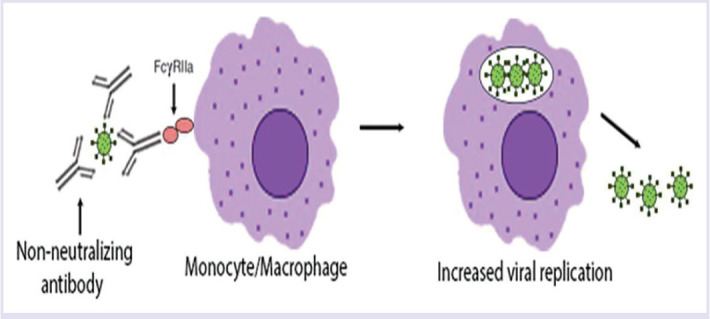
ADE of infection is shown. The mechanism includes IgG antibody Fc-region binding to the Fcγ receptor on an immune system cell, the Fcγ receptor functionally imitates the actual viral receptor, and therefore facilitation of viral entry and then the conclusion of increased viral replication. ADE: Antibody-dependent immune enhancement.

Antibody covered virus is normally attracted into cells carrying Fcγ receptors, involving monocytes and macrophages [56]. ADE is described as either the acceleration of viral access into the cell by antibody or the augmentation by virally toxic antibody. It is theorized that the mechanism includes IgG antibody Fc-region tied to the Fcγ receptor on any immune system cells, such that the Fcγ receptor practically imitates the real viral receptor and, thus, facilitates viral access (Fig. 1). ADE is usually supposed to happen when antibody levels are deficient to completely prevent viral entry but are adequate to opsonize virus. It is also thought that antibodies produced throughout previous infection with a dissimilar viral serotype could aggravate clinical severity of the present disease [6, 55]. CIP might also cause the direct transfusion of a large quantity of complement proteins and coagulation elements not detected in refined immunoglobulin products. A further distress concerning transfusions of complement comes from investigations in other infections such as HIV and Ebola where complement-dependent antibody enhancement has been shown [56].

Despite a hypothetical possible risk of ADE of infection, there have been no descriptions of this incidence happening with the SARS-CoV-1 or MERS viruses by means of CIP administration. This was observed with dengue virus, amid other viral diseases [4]. However, detailed investigations to evaluate this possible risk are needed, specifically concerning vaccine policy and utilization of monoclonal antibody-based treatment in COVID-19.

## Immediate Intravascular Hemolytic Transfusion Reactions (IIHTRs)

IIHTRs are thought to be the most severe obstacle of ABO incompatible plasma administration. Nevertheless, the possibility of IIHTR taking place during a transfusion is predicted to be between 1:2000 and 1:9000 in cases with serious systemic disorders, and mortality is exceptionally infrequent. It is prudent to keep away from the transfusion of blood group O plasma to patients with group AB. Quantitative examination of anti-A and anti-B titers should be measured for expecting hemolysis. This isohemagglutination test is a technique for the assessment of immunoglobulin M levels, with a titer above 1:100 demonstrating a risk for hemolysis [55, 57].

## Cautionary Points in the CIP Treatment

• It is vital to confirm ABO blood group matching of plasma between the donor and the recipient [31].
• Pre-treatment to diminish transfusion-associated events might be thought [7].
• Cases at risk of TACO should be transfused gradually [7].
• CIP transfusion from no <2 donors might be therapeutically valuable to accomplish more efficient immune defense by means of gaining assorted antibodies [31].
• Cases can be given a preliminary transfusion of 200 mL, subsequently one or two extra transfusions of 200 mL depending on disease seriousness and tolerance to the transfusions [31].
• COVID-19 cases having cytokine storm syndrome would not donate CIP after improvement [35].
• A recovering individual had better give CIP only once [35].

## Contraindications of CIP

Explicit contraindications consist of: (1) a history of allergic reaction to plasma, (2) a history of autoimmune systemic disorder, or (3) selective IgA deficiency. In these individuals, the administration of CIP should be assessed carefully by the physician [23].

## Pearls of CIP Treatment

• High-titer specific antibodies to attach to SARS-CoV-2 virus and deactivate the viral particles [58]
• Shown effectiveness in past viral infections and SARS-CoV-2 outbreaks [58, 59]
• It is best when transfused in the earlier phase of COVID-19 disease
• Few significant secondary modulatory effects [58]

## Pitfalls of CIP Treatment

• Expensive [58]
• Arduous logistics [58]
• Potential administration in severe, treatment refractory cases [2]
• Short of high-quality investigations, that is, randomized controlled trials [58, 60]
• Insufficient information on the essential biology of SARS-CoV-2, plus its unpredictability and mutations [58]
• The quantity of NAT transfused to each individual was unidentified and not consistent, which may lead to disparity in clinical result [37]
• A type of passive immunization, consequently a short period of the effect [58, 60]
• It still has its hidden risk of exaggerating hyperimmune attack [8]
• It is uncertain whether CIP can cloud the progress of an innate immunological reaction, particularly when utilized preventatively [31].

## Conclusion

The recent studies suggest that CIP transfusion can assist to impede viral spread and improve survival in COVID-19 cases having pulmonary insufficiency, although it could not significantly decrease the fatality rate in seriously ill cases with end-stage disease. Reliant on the recent findings, CIP therapy should be started to COVD-19 cases at the right time point and should probably be utilized in potentially seriously ill individuals at an early phase of COVD-19. CIP may have a significant role as one of the therapeutic modalities for various viral infections when enough vaccines or other specific therapeutic agents are not on hand [61].
